# Efficacy and mechanisms of combined aerobic exercise and cognitive training in mild cognitive impairment: study protocol of the ACT trial

**DOI:** 10.1186/s13063-018-3054-0

**Published:** 2018-12-22

**Authors:** Fang Yu, Feng Vankee Lin, Dereck L. Salisbury, Krupa N. Shah, Lisa Chow, David Vock, Nathaniel W. Nelson, Anton P. Porsteinsson, Clifford Jack

**Affiliations:** 1 University of Minnesota School of Nursing, 5-160 WDH 1331, 308 Harvard St SE, Minneapolis, MN 55455 USA; 2 University of Rochester Medical Center, 601 Elmwood Ave, Rochester, NY 14642 USA; 3 University of Minnesota School of Medicine, Minneapolis, MN USA; 4 University of Minnesota Division of Biostatistics, Minneapolis, MN USA; 5 University of St. Thomas, Minneapolis, MN USA; 6 Department of Radiology, Mayo Clinic, Rochester, MN USA

**Keywords:** Aerobic exercise, Alzheimer’s disease, Cognitive training, Neuroimaging, Executive function, Memory, Aerobic fitness

## Abstract

**Background:**

Developing non-pharmacological interventions with strong potential to prevent or delay the onset of Alzheimer’s disease (AD) in high-risk populations is critical. Aerobic exercise and cognitive training are two promising interventions. Aerobic exercise increases aerobic fitness, which in turn improves brain structure and function, while cognitive training improves selective brain function intensively. Hence, combined aerobic exercise and cognitive training may have a synergistic effect on cognition by complementary strengthening of different neural functions. Few studies have tested the effects of such a combined intervention, and the findings have been discrepant, largely due to varying doses and formats of the interventions.

**Methods/design:**

The purpose of this single-blinded, 2 × 2 factorial phase II randomized controlled trial is to test the efficacy and synergistic effects of a 6-month combined cycling and speed of processing training intervention on cognition and relevant mechanisms (aerobic fitness, cortical thickness, and functional connectivity in the default mode network) in older adults with amnestic mild cognitive impairment. This trial will randomize 128 participants equally to four arms: cycling and speed of processing, cycling only, speed of processing only, or attention control for 6 months, and then follow them for another 12 months. Cognition and aerobic fitness will be assessed at baseline and at 3, 6, 12, and 18 months; cortical thickness and functional connectivity at baseline and at 6, 12, and 18 months; Alzheimer’s disease (AD) conversion at 6, 12, and 18 months. The specific aims are to (1) determine the efficacy and synergistic effects of the combined intervention on cognition over 6 months, (2) examine the underlying mechanisms of the combined intervention, and (3) calculate the long-term effect sizes of the combined intervention on cognition and AD conversion. The analysis will use intention-to-treat and linear mixed-effects modeling.

**Discussion:**

This trial will be among the first to test the synergistic effects on cognition and mechanisms (relevant to Alzheimer’s-associated neurodegeneration) of a uniquely conceptualized and rigorously designed aerobic exercise and cognitive training intervention in older adults with mild cognitive impairment. It will advance Alzheimer’s prevention research by providing precise effect-size estimates of the combined intervention.

**Trial registration:**

ClinicalTrials.gov, NCT03313895. Registered on 18 October 2017.

## Background

Preventing Alzheimer’s disease (AD) is the most important approach for tackling the AD epidemic. In 2018, AD afflicts 5.7 million Americans at a cost of $277 billion and up to 14 million Americans by 2050. In 2017, 16.1 million American family caregivers provided 18.4 billion hours of unpaid care valued at $232 billion [1]. However, no drugs can yet prevent, treat, or even slow down the progression of AD [2]. Mild cognitive impairment (MCI) is the prodromal stage of AD with well-defined diagnostic criteria and phenotype [3], representing a critical opportunity for AD prevention. MCI has an annual incidence rate of 51–77 per 1000 [3]. For amnestic MCI (aMCI), there is a cumulative 32% conversion rate to AD or other dementia [4]. Individuals with MCI are highly motivated to engage in interventions to prevent further cognitive decline [5, 3]. Currently, aerobic exercise and cognitive training are the two most promising non-pharmacological interventions for preventing conversion from MCI to AD [6].

Aerobic exercise refers to physical activity that uses large muscle groups to maintain continuous and rhythmic movements to improve aerobic fitness (the ability of the heart to deliver oxygen to working muscles), which is measured by peak oxygen consumption (VO_2peak_) [7]. Epidemiological evidence has consistently supported exercise’s link to reduced AD risk [8–12]. Randomized controlled trials (RCTs) have demonstrated that aerobic exercise such as cycling produced modest to moderate gains across cognitive domains in persons with intact cognition [13–16], MCI [17, 18], or dementia [15, 19]. The effect of aerobic exercise on cognition or AD risk may be explained by the relationship between aerobic fitness and brain integrity. High aerobic fitness has been associated with less brain atrophy [20–24] and stronger functional connectivity [25–28] across the brain in older adults with intact cognition or early AD.

Cognitive training involves repeated practice of a set of standard cognitive tasks targeting specific cognitive domains [29]. Targeting cognitive processes (e.g., attention) may produce more robust and broadly generalizable effects than targeting cognitive structures (e.g., delayed recall) [29–31]. Previous studies found that such process-based cognitive training generated a moderate to large improvement in selected cognitive domains in older adults with intact cognition or MCI. For example, attention training, such as speed of processing (SOP) training, has positive effects on sustained and divided attention [32], perfusion of the prefrontal cortex, and the functional connectivity between the occipitotemporal areas [33, 34] as well as within the default mode network (DMN) [35].

The preceding evidence indicates that aerobic exercise and cognitive training affect brain structure and function as well as cognition via distinct mechanisms. Combined Aerobic exercise and Cognitive Training (ACT) may build upon the strength of each intervention while concomitantly offsetting its relevant weakness to maximize the effects of these interventions for slowing the progression of AD, essentially due to their additive/synergistic neural effects. In other words, aerobic exercise that occurs in a cognitively challenging environment may produce greater cognitive and neural benefits than exercise alone [36–39]. Animal research indicates that aerobic exercise causes a large increase in new neurons, but at least half of them undergo apoptosis within weeks [40, 41]. Fortunately, these neurons can be rescued to become functional if trained with challenging cognitive tasks over time [36, 39, 41]. Aerobic fitness may also strengthen the neural plasticity obtained from cognitive training [40]. In humans, ACT intervention studies are few, with discrepant findings due to different intervention components, doses, and delivery formats [42–46]. Historically, ACT has been examined in two formats: concurrent (e.g., exergames, combining physical exercise with virtual reality training) vs. sequential (e.g., one after another). Whereas the exercise components can be comparable, the two formats differ considerably in the cognitive component (virtual reality such as scenery vs. cognitive training). Furthermore, the exact design of the sequential format lacks clarity. For example, a study using sequential design did not specify how the two components were delivered (e.g., on the same day or on different days; if on the same day, the amount of time lapse between the two components) [42]. A recent study of ACT failed to find a significant effect on gray matter in MCI, which may also be due to the lack of consideration of the intervention format [47]. Hence, there is a critical need to develop different delivery formats of ACT interventions that are maximally potential and implementable and investigate their effects and mechanisms of action. Such studies are best addressed by phase II trials which are also considered stage II trials according to the National Institutes of Health (NIH) Stage model [48].

### Study aims

The purpose of this phase II RCT is to test the efficacy and additive/synergistic effects of an ACT intervention (cycling and SOP training), by comparing it to cycling and SOP training alone and an attention control (stretching and mental leisure activities), on cognition and relevant mechanisms (aerobic fitness, AD signature cortical thickness, and DMN) in older adults with amnestic MCI (aMCI). Here we choose AD signature cortical thickness [49] and DMN [50] since they are common neural markers detected early in the progression of AD. The study is funded by the NIH’s National Institute on Aging (R01AG055469-01A1, 9/15/2017–5/31/2022). The specific aims and hypotheses of the study are described as follows:

- Aim I. Determine the efficacy and additive/synergistic effects of ACT on cognition over 6 months

*Hypothesis 1*. ACT will have the greatest effects on executive function and episodic memory compared with other groups.

- Aim II. Examine the underlying mechanisms of ACT over 6 months

*Hypothesis 2a*. ACT will have the greatest effects on AD signature cortical thickness, functional connectivity in the DMN, and aerobic fitness compared with other groups.

*Hypothesis 2b*. Changes in the mechanistic measures are related to cognitive changes.

*Hypothesis 2c*. Changes in AD signature cortical thickness and DMN mediate aerobic fitness effects on cognition.

- Aim III (exploratory). Calculate the long-term effect sizes of ACT on cognition and clinical AD conversion to inform future phase III RCTs

## Methods

### Design

**Fig. 1:**
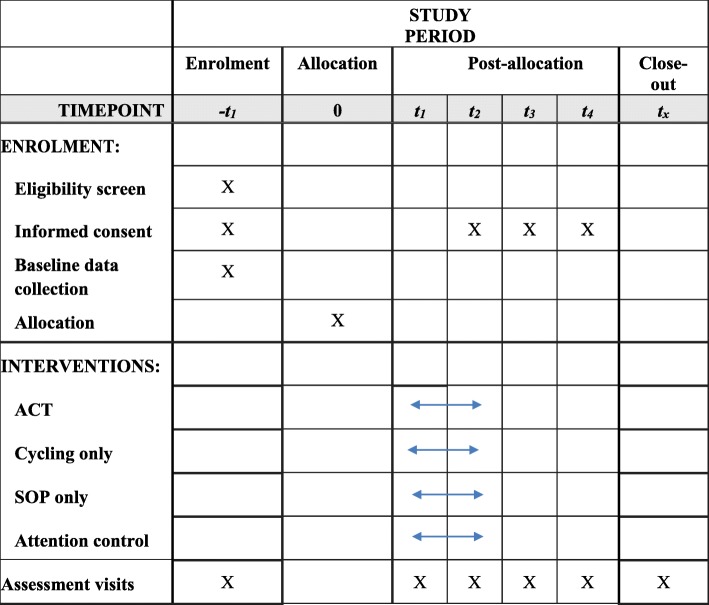
Schedule of enrollment, interventions, and assessments

This RCT will use a single-blind, multi-site, 2 × 2 factorial design, guided by the Consolidated Standards of Reporting Trials (CONSORT) [51] and the Standard Protocol Items: Recommendations for Interventional Trials (SPIRIT) checklist [52]. We will randomize 128 older adults with aMCI equally to one of four arms for 6 months (*n* = 32 in each group): ACT; cycling only; SOP training only; or attention control. Based on our previous studies (Fig. 1) [35, 53, 54], we anticipate screening approximately 384 individuals in person to enroll 128 participants. Participants will then be followed for another 12 months (*n* = 96 at 6 months with 25% attrition and *n* = 84 at 18 months with 35% attrition). We will stratify randomization by site (University of Minnesota [UMN] and University of Rochester [UR]) and age (< 75 and ≥ 75 years), using random permuted blocks of 4 and 8 participants. Cognition and aerobic fitness will be assessed at baseline and at 3, 6, 12, and 18 months; AD signature cortical thickness and DMN at baseline and at 6, 12, and 18 months; AD conversion at 6, 12, and 18 months.

### Setting

Screening and non-imaging data collections will occur in a private room at the Clinical and Translational Science Institute and Laboratory of Clinical Physiology at UMN and the CogT Lab at UR. Imaging will occur at the Center for Magnetic Resonance Research at the UMN and the Rochester Center for Brain Imaging at the UR. Both centers have participated in multi-site imaging studies with harmonized MRI protocols previously. The interventions will occur at a local YMCA gym or senior center close to the participant’s residence.

### Study population

#### Recruitment

We plan to enroll 128 participants within 3.5 years (4–5 participants a month) with a proactive recruitment plan, including clinic referrals, recruitment material distributions in the communities, exhibits at relevant conferences, and media such as newspaper advertisements. We will emphasize minority recruitment by educating providers serving minorities, collaborating with community gatekeepers, and hiring specialists for minority recruitment. In the event that recruitment goals are not being met, we will consider zip code mass mailing, initiate recruitment from other health systems outside the two universities, and reach out to suburban and rural neighborhoods.

#### Eligibility and screening

Potential participants who respond to our recruitment strategies and initiate contact with us will be carefully screened for eligibility and safety using a three-step procedure, including a phone interview, an in-person interview, and a symptom-limited peak cycle-ergometer test and magnetic resonance imaging (MRI) scan to ensure they meet our eligibility criteria (Table 1).

1. Phone interview: The staff will get the respondent’s verbal consent for participating in the phone and in-person screenings. The staff will elicit self-reported health history and exercise/MRI risk. Potential participants who pass the phone screen will be scheduled for an in-person interview.
2. In-person interview: Informed consent and Health Insurance Portability and Accountability Act (HIPAA) authorization will be obtained. The study staff will corroborate phone screen data, administer instruments to assess clinical diagnosis of aMCI, conduct a focused physical assessment, and complete the MRI safety checklist. During informed consent, individuals will be informed about the study procedure, data collection, time commitment, the voluntary nature of study participation, risks, benefits, compensations, and contact within and outside the study team for human subjects. Letters will be sent to the potential participant’s medical providers to obtain medical clearance for exercise and MRI. Medical clearance from a cardiologist will also be obtained if a participant has a significant cardiac history and has a cardiologist. The investigator team will make safety decisions for those who do not have a cardiologist.
3. Symptom-limited peak cycle-ergometer test and MRI: Upon receiving safety clearance for exercise, potential participants will undergo a symptom-limited peak cycle-ergometer test to further rule out unknown cardiac ischemia and serious arrhythmia and to obtain peak heart rate (HR) for calculating HR reserve. Resting seated and standing HR, blood pressure, and electrocardiograms will be evaluated to ensure safety prior to performing the test. HR and rhythm will be continuously monitored during exercise, and blood pressure will be monitored every 3 min during the test. The test will be stopped if any of the American College of Sports Medicine (ACSM) indications for absolute or relative test termination are met. The test will be conducted in the exercise labs by trained lab technicians, interventionists, or their designees. The supervising clinicians will determine if the criteria for ending the test are met, or if the test needs to be terminated, and whether participants can proceed towards enrollment. Participants who pass the symptom-limited peak cycle-ergometer test and have no contraindications for the MRI will undergo a 30-min scan to see if they any have brain abnormalities that are clinically significant to warrant a follow-up medical evaluation and/or would interfere with a research definition of MCI such as normal pressure hydrocephalus, brain tumor, subdural hematoma, significant post-traumatic encephalomalacia, or one or more large hemispheric infarctions. Participants with clinically significant brain abnormalities will be referred to their primary care providers for medical follow-up and will be considered for study participation only if the providers medically clear them. Those who have no brain abnormality and those who have clinically significant brain abnormalities but have been medically cleared will be formally enrolled in the study upon completing baseline data collection and randomized after baseline data collection.

**Table 1:** Eligibility criteria

Inclusion criteria	Exclusion criteria
a) A clinical, *consensus* diagnosis of aMCI (mono-domain or multi-domain): ◦ 18 ≤ Montreal Cognitive Assessment score ≤ 26 (education-corrected) ◦ Memory deficits: at least 1 standard deviation below age- and/or education-corrected population norms on Rey Auditory Verbal Learning Test ◦ Preserved activity of daily living: Activities of Daily Living-Prevention Instrument score < 30 ◦ Absence of dementia ◦ A consensus clinical diagnosis of MCI (using 2011 Alzheimer’s association-NIA criteria) by the investigators b) Community-dwelling, e.g., homes and assisted living c) Age 65 years and older d) English-speaking e) Adequate visual acuity for testing f) Verified exercise safety g) Stable on drugs affecting cognition > 3 months, if on these drugs h) Verfied MRI scan safety i) Capacity to consent	a) Geriatric Depression Scale score > 5, with contextual evidence suggesting unstable major depression or psychiatric disorders such as taking antidepressant less than 3 months b) Resting HR ≤ 50 due to arrhythmia or ≥ 100 beats/min c) Neurologic, psychiatric, or substance abuse disorders in past 5 years that are the main contributor to MCI d) ACSM contraindications to exercise e) New, unevaluated symptoms or diseases f) Current enrollment in another intervention study g) Abnormal MRI findings

*ACSM* American College of Sports Medicine, *HR* heart rate, *MCI* mild cognitive impairment, *MRI* magnetic resonance imaging, *NIA* National Institute on Aging

#### Sample size and power

We determined the sample size based on anticipated effect sizes, attrition, and the literature. For Aim I, a sample size of 128 at 25% attrition will give us a final analytical sample of 96 at 6 months. Our previous study of SOP training had a partial η^2^ = 0.28 for working memory [35], while cycling had a value *r*^2^ = 0.35 for slowing the worsening of ADAS-Cog (the Alzheimer’s Disease Assessment Scale-Cognition, a global cognition measure [55]. Here, with an estimated, conservative effect size for the change in cognition for ACT (compared with attention control) of 0.25, we will have 80% power to detect group differences in both episodic memory and executive function. For Aim II, our previous study of SOP training had a partial η^2^ = 0.62 for mechanistic effect [35]. Using a type I error rate of 0.05, up to three repeated measures (0, 3, and 6 months), and a correlation between measures of 0.5, we only need 58 participants to have 86% power to detect a significant difference in the change in AD signature cortical thickness and DMN with an effect size (partial η^2^) of 0.08. Aim III is exploratory and not powered. A 35% attrition at 18 months will result in a final analytical sample of 84 to calculate ACT’s long-term effect sizes to inform future RCTs.

### Variables and their measures

#### Primary outcomes

The primary outcomes for this RCT include cognition, AD signature cortical thickness, functional connectivity in DMN, aerobic fitness, and clinical AD conversion. Cognition and aerobic fitness will be assessed at baseline and at 3, 6, 12, and 18 months; AD conversion (including reversion to normal cognition) at 6, 12, and 18 months; and AD signature cortical thickness and DMN at baseline and at 6, 12, and 18 months (Table 2).

**Table 2:** Data collected from participants over time (S: Screening)

Variables	Measures (data types)	Source of data collection	S	Month				
				0	3	6	12	18
Cognitive outcomes								
Executive function	EXAMINER (continuous)	Computer-based and paper based test		x	x	x	x	x
Episodic verbal memory	RAVLT (continuous)	Pencil-paper-based test	x		x	x	x	x
Episodic visual memory	BVMT-R (continuous)	Pencil-paper-based test		x	x	x	x	x
AD conversion-clinical	Consensus diagnosis using 2011 criteria for probable AD (categorical)	All data collected				x	x	x
MCI reversion	ADNI criteria (categorical)	All data collected				x	x	x
MRI mechanistic outcomes								
Cortical thickness	Structural MRI (continuous)	Device measured	x			x	x	x
Functional connectivity in DMN: resting state	fMRI (continuous)	Device measured	x			x	x	x
Aerobic fitness outcomes								
Aerobic fitness	Symptom-limited peak cycle-ergometer test (continuous)	Performance-based test	x		x	x	x	x
Aerobic fitness	10-m ISWT (continuous)	Performance-based test		x	x	x	x	x
Other variables								
Implementation consistency	Consistency checklist (continuous)	REDCap report	Monthly					
Global cognition	MoCA (continuous)	Pencil-paper-based test	x		x	x	x	x
Daily functioning and mood	ADL-PI, WHODAS (all continuous)	Self-report, paper form	x		x	x	x	x
Depression	GDS (continuous)	Self-report, paper form	x	x	x	x	x	x
Medical changes	Medical diagnoses, falls, medications, and health service use (all categorical)	Self-report, paper form	Monthly					
Demographics	Age, education (both continuous), gender, race (both discrete)	Self-report, paper form	x					
Premorbid intellect	WTAR (continuous)	Pencil-paper-based test	x					
Non-study physical and mental activities	Wearable activity monitor, PASE questionnaire, MLA monthly (continuous)	Wrist device; pencil-paper-based questionnaire	Monthly					
Adherence	Percent (continuous)	Paper form	Monthly					

Tests administered during screening will be used as baseline data to avoid practice effects

*AD* Alzheimer’s disease, *ADL-PI* Activities of Daily Living-Prevention Instrument, *ADNI* Alzheimer’s Disease Neuroimaging Initiative, *BVMT-R* Brief Visuospatial Memory Test-Revised, *ISWT* Incremental Shuttle Walk Test, *MCI* mild cognitive impairment, *MLA* mental leisure activity, *MoCA* Montreal Cognitive Assessment, *MRI* magnetic resonance imaging, *PASE* Physical Activity Scale for the Elderly, *RAVLT* Rey Auditory Verbal Learning Test, *WHODAS* World Health Organization Disability Assessment Schedule, *WTAR* Wechsler Test of Adult Reading

#### Cognition

Cognition includes composite scores for executive function and episodic memory, respectively. Executive function will be measured using the EXAMINER, a computerized test package designed for RCTs. It calculates three subdomain composite scores on working memory, cognitive control, and fluency and an overall composite score for executive function [56]. Test-retest reliabilities are .78 to .93 [57]. EXAMINER has three alternative packages to reduce practice effects. Episodic memory will be assessed using the Rey Auditory Verbal Learning Test (RAVLT) [58] and the Brief Visuospatial Memory Test-Revised (BVMT-R) [59]. These tests are similar in format; however, one utilizes verbal learning while the other utilizes visual learning, allowing a comprehensive assessment of visual and verbal memory. A composite score combining immediately and delayed recalls from the two tests will be developed.

#### AD signature cortical thickness and DMN

##### MRI harmonization

Both sites will use 3 T Siemens Prisma scanners (Erlangen, Germany). Quality-control checks on the phantom, volunteer data, and imaging sequences will be conducted before any data collection. MRI data will be analyzed at the CogT Lab at UR.

##### MRI data acquisition

Structural MRI, fluid-attenuated inversion recovery (FLAIR), and blood oxygen level-dependent (BOLD) functional MRI (fMRI) data will be collected in a 30-min session. Each session will begin with a localizer scan, followed by a magnetization-prepared rapid gradient echo imaging (MPRAGE) scan with 1-mm isotropic resolution to provide high-resolution structural-weighted anatomical images. Three-dimensional FLAIR and susceptibility weighted images (SWIs) will be acquired to ascertain brain pathology (e.g., cerebrovascular disease). Field maps will be acquired to correct for distortions in echo-planar imaging sequences. BOLD functional data during resting state will be collected using a gradient echo-planar imaging sequence. The resting-state scan will be acquired using simultaneous multi-slice imaging. Quality control of each MRI session will be performed.

##### Structural MRI data preprocessing and analysis

We will use the structural MRI measure to develop a composite score of AD signature cortical thicknesses segmented and analyzed with FreeSurfer, composing the following individual regions of interest: precuneus, fusiform, and inferior and middle temporal lobes [49].

##### fMRI data preprocessing and analysis

The data will be analyzed using the SPM software package. fMRI preprocessing will consist of motion correction, slice-timing correction, normalization, and Gaussian spatial smoothing. All the regions of interest in the DMN will be extracted based on our and others’ independent component analysis results [50]. To calculate resting-state fMRI, the correlation between each selected region pair will be Fisher Z-transformed and averaged to derive a summary score for DMN strength.

#### Aerobic fitness

Aerobic fitness will be measured as peak oxygen consumption (VO_2peak_) from the symptom-limited peak cycle-ergometer test and the 10-m Incremental Shuttle Walk Test (ISWT) [60]. The participant will begin cycling at a comfortable speed, then encouraged to increase the intensity as the resistance is increased every 3 min (1 metabolic equivalent, = 3.5 ml oxygen/kg body weight/minute). The participant will continue until fatigued or until the test termination criteria are satisfied per the ACSM. VO_2peak_ and peak hemodynamic responses (e.g., HR, heart rhythm, blood pressure) will be continuously monitored via electrocardiogram and indirect calorimetry. The Borg Rating of Perceived Exertion (RPE) will be assessed during the last minute of each stage and at peak exercise. The ISWT will be used as a field test for the evaluation of aerobic fitness. The distance covered in the ISWT has shown correlations with VO_2peak_ obtained from graded exercise tests (*r* = .67–.95) [60].

#### AD conversion or reversion to normal

AD conversion is defined as meeting clinical criteria for AD. Beginning with the 6 months assessment, a consensus clinical diagnosis of AD will be determined using the 2011 probable AD diagnostic criteria [61]: memory deficit (≥ 1.5 standard deviation (SD) below age- and education-corrected norms), Montreal Cognitive Assessment (MoCA) < 18, decline in activities of daily living, cognitive changes not due to other conditions, and observed pathology (e.g., degree of atrophy). MCI reversion to normal cognition will be assessed and treated similarly as AD conversion. At the 18 months follow-up, a final classification will be determined for each participant (e.g., constant as MCI, conversion to AD, reversion to normal) based on the AD Neuroimaging Initiative criteria [62].

#### Covariates

Covariates include implementation consistency, global cognition, medical conditions, non-study physical and mental leisure activities (MLAs), premorbid intellect, and adherence. Covariates were selected for their known relationship with cognition and will be measured with psychometrically sound instruments, but they are not all inclusive since randomization will minimize imbalances among groups.

Implementation consistency between sites will be measured using four indicators: participant accruals (number of respondents, enrolled and per group); baseline characteristics (overall and in each group); adherence to the assigned activity and reasons for not adhering; and time to reach key milestones (e.g., initial contact to each screening step, enrollment to baseline, baseline to first activity session, and duration for completing assigned activity). Global cognition will be measured using MoCA. Medical conditions will be assessed using the Medical Changes, Health Service, and Falls Assessment Form, and will include diagnoses, falls, medications, and health service use. Depression will be measured by the Geriatric Depression Scale (GDS). Non-study physical and mental activities will be measured by the MLA questionnaire, a wearable activity monitor, and the Physical Activity Scale for the Elderly (PASE) questionnaire. Daily functioning and mood will be measured using the Activities of Daily Living-Prevention Instrument (ADL-PI, self-administered) and the WHO Disability Assessment Schedule (WHODAS). Premorbid intellect will be assessed using the Wechsler Test of Adult Reading (WTAR).

### Study procedure

#### Study preparation and randomization

We will develop a Study Operations Manual to ensure implementation consistency across sites. All staff will be adequately trained to ensure participant safety, blinding, data quality, and protocol adherence. Prior to any recruitment, the statistician will create a randomization schedule in REDCap using a random number generator. Qualified participants will be randomized after completing baseline data collection. The study staff (coordinator/interventionist) will log into the REDCap randomization module and enter a participant’s name/site/age. The randomization module will assign the participant to a group and record the assignment in a database that is only accessible to these staff.

#### Data collection

Enrolled subjects will be scheduled to complete baseline data collection. Subsequent data collection will occur at 3, 6, 9, and 12 months for cognition and other outcomes which are further described under “Variables and their measures” and in Fig. 1. Subjects who undergo MRI at baseline will be re-evaluated for eligibility for MRI at 6 and 12 months (Fig. 1). To ensure the blindness of the data collectors from the intervention assignment, the data collectors will not interact with enrolled subjects except for data collection.

#### Delivery of the assigned activity for each group

Within 2 weeks of completing baseline data collection, participants will start their assigned activity: ACT, cycling only, SOP training only, or attention control. Each activity will consist of a total of three weekly, supervised sessions for 6 months (72 total sessions). One interventionist will supervise two to three participants in the same activity. The sessions will be delivered over up to a 28-week period to account for missing sessions due to the data-collection week at 3 months, vacations, and illnesses.

##### Cycling only

Moderate-vigorous intensity will be prescribed using an undulating (non-linear) progression scheme with intensity ranges from 50 to 75% of HR reserve and/or 11–15 on the RPE scale and exercise durations of 30–50 min per session. HR reserve will be calculated by subtracting resting HR (after 10 min of quiet resting) from the peak HR achieved during the symptom-limited peak cycle-ergometer test. Cycling will be set at 50–60% of HR reserve or RPE 11–12 for 30 min in session 1, and will be alternatively increased by 5% of HR reserve (or 1 point on Borg) or 5-min increments as tolerated up to 65–75% of HR reserve (or RPE 13–15) for 50 min per session over time. Participants will do a 5-min cardiac warm-up and cool-down before and after cycling following ACSM guidelines. In each session, the interventionists will equip the participants with wireless HR monitors, take resting HR and blood pressure, and monitor HR, RPE, talking ability, and signs and symptoms of overexertion every 5 min, and blood pressure every 10–15 min. Participants may leave after their HR and blood pressure return to pre-cycling levels. Each month, participants will wear a device on their wrists for 7 consecutive days to track study-unrelated physical activity. The interventionists will help put the device on at the end of a session and take it off before the activity session on day 7.

##### SOP training only

SOP training will involve the use of the InSight online program (Posit Science). This includes five games (Eye for Detail, Hawk Eye, Visual Sweeps, Double Decision, and Target Tracker) that involve multiple cognitive processes, primarily in attention and processing speed. All games share visual components, and the tasks become increasingly difficult and require faster reaction times as they progress. Participants will respond either by identifying what object they see or where they see it. The training will automatically adjust the difficulty of each task based on performance, ensuring that participants always operate near their optimal capacity. The training programs will automatically record the percentage of completion of each game and the scores. The initial training will take longer to allow participants to adapt to the style of cognitive training. Gradually, individuals will require a shorter practice time to retain the training effect. The interventionists will supervise and help with technical problems as needed. Training will occur in a semi-private room, where each participant will have his own workspace, computer, and headset to avoid distraction.

##### ACT

Participants will participate in cycling first and SOP training second without time lapse in each session. Session durations for ACT will be 80 min (sum of session durations for cycling only and SOP training only).

##### Attention control

Participants will participate in stretches and MLAs that we have previously tested and that are matched to session durations for cycling and SOP training, respectively. Stretching includes seated movements and static stretches that induce no changes in aerobic fitness [63]. MLAs include online word search, Sudoku, and solitaire games that are similar to everyday mental activities without the SOP training’s time, speed, or novel cognitive stimuli elements, which are essential for improving cognition [35, 63]. These activities will control for social interaction, computer, and online experience effects of the ACT and increase participant retention.

##### Treatment fidelity

In a multi-site study, ensuring implementation consistency across sites is of paramount importance to reach valid results. This study was designed to ensure treatment fidelity based on the NIH Behavior Change Consortium recommendations [64] and our experience [65]. We will randomly check activity sessions in person using the Fidelity Checklist and retrain staff as needed.

##### Safety, retention, adherence, and validation

We have built in many strategies tested in our preliminary studies to protect participants against risks, including appropriate equipment and personnel training, careful screening and informed consent, frequent communications, individualized prescriptions for cycling and SOP training, supervised and guided sessions, and ongoing monitoring of medical changes. Interventionists will be fully trained using the operations manual and will be able to consult with the investigators immediately as well as at weekly meetings. Our previous work with AD and aMCI achieved 74.3–86.4% adherence to cycling [65] and 100% adherence to SOP [35]. The safety strategies will also ensure retention. For this RCT, we predicted a higher attrition rate (25% at 6 months and 35% at 18 months) than those of our current RCTs with AD and aMCI (4.3–11.1% attrition) [35, 65].

### Data management

#### Data entry, coding, and storage

The principal investigators (PIs) will be responsible for the integrity and validity of the study data. The PIs and the study coordinators will oversee all participant records, adherence to the protocol by all staff, screening, and recruitment logs, percent of exercise sessions attended, missed sessions, compliance, retention, adverse events, and study outcomes. They will also ensure data creation, completeness, quality checks, and audits. The PIs and statistician will develop a data audit to ensure data accuracy and completeness. Participant data will be collected with the verbal or written consent of the participant. Information pertaining to individual participants will be released with the participant’s permission only. All participant data will be identified by a uniquely coded screening ID assigned to each respondent and a study ID for participants eligible for enrollment. Access to the master links between name and screening ID as well as between screening ID and study ID will be restricted to the study coordinator, interventionist, and staff involved in screening.

Data collection forms returned to the research office by the research assistants (RAs) will be reviewed by the study coordinators for accuracy and completeness before entry into REDCap, a MySQL database accessed via a secure web interface. The MySQL database and the web server will both be housed on secure servers operated by the UMN Acadmic Health Center-Information Systems (AHC-IS) or UR Information Technology Services (ITS). The servers are in a physically secure location on campus and are backed up nightly, with the backups stored in accordance with the retention schedule of daily, weekly, and monthly tapes retained for 1, 3, and 6 months, respectively at each university’s information services. Weekly backup tapes are stored offsite. The AHC-IS or ITS servers provide a stable, secure, well-maintained, and high-capacity data storage environment that meets the requirements for storing HIPAA-sensitive data, and both REDCap and MySQL are widely used, powerful, reliable, well-supported systems. Access to the system by username and password will require specific permission from the respective PI or her designee. The PIs and study coordinators will continually work with the study sites to ensure compliance and quality. The PIs will verify data accuracy and completeness in REDCap for data collected at each site. Any missing data will be assigned to a data collector for collection. If a participant withdraws from the study, all attempts will be made to collect data to allow for inclusion in the analysis. Reasons for withdrawal will be recorded.

Data audits of electronic outcome data will be conducted by the PIs and study coordinators. If the PI identifies any discrepancies in scoring between the RA who collected data and the RA who entered data, the RAs must consult the neuropsychologist co-investigator for correct scoring, and the correct scores will be documented on paper and entered into REDCap.

#### Data Safety and Monitoring Board (DSMB)

The DSMB comprises three senior scientists with expertise in geriatric medicine, exercise, or cognitive training, or RCTs external to the UMN and UR and vetted by the National Institute on Aging (NIA). The DSMB ensures participants’ safety and the integrity and validity of the collected data. One DSMB member will be assigned the role of Chair and Safety Officer. During the project period, DSMB members will have no direct involvement with the study. The chairperson will be responsible for overseeing the meetings and developing the agenda in consultation with the NIA Program Official and the PIs. The DSMB will monitor and review participant safety and data confidentiality, participant accrual and retention, adherence to inclusion and exclusion criteria, adverse events, the quality of data collection, management, and analysis, and study progress; this information will be submitted to the NIA. The DSMB will also monitor the ongoing integrity of the study and review any preliminary data analyses and annual reports submitted by the PIs to the respective Institutional Review Board (IRB) and NIH offices for continuing protection of human participants. The PIs will be informed of serious adverse events as soon as they occur and will notify the IRB, DSMB, and NIA within 5 business days. The DSMB will meet either in person or by teleconference initially following enrollment of 10% of participants and semi-annually after its first meeting.

### Statistical analyses

All variables will be summarized using appropriate descriptive statistics (e.g., means/SD for continuous measures, and frequencies for categorical variables). Baseline variables will be compared among the four groups to determine systematic differences using Pearson’s chi-square test (categorical variables) and analysis of variance (ANOVA) or Kruskal-Wallis (continuous variables), as appropriate. Variables that differ significantly among groups will be included as covariates in the models described below. All analyses will follow the intention-to-treat principle (e.g., group assignment in the analysis will be based on randomized group assignment regardless of level of adherence to provide unbiased comparisons of the effects among the groups). This principle will account for any potential data loss. Because Aims I and II focus on cognition and mechanisms, they will be analyzed regardless of MCI clinical phenotype. We will also compare implementation consistency between sites using Pearson’s chi-square test.

#### Aim I. Determine the efficacy and additive/synergistic effects of ACT on cognition over 6 months

We will fit linear mixed-effects models for the change in episodic memory and executive function, accounting for repeated measures at baseline and at the 3 months and 6 months visits (the data for 12 and 18 months will be used in the exploratory aim (Aim III)). Fixed effects in the model will include data collection visit (categorical variable), group assignment, and visit and group interaction. Covariates identified as being imbalanced among groups may be included in the analyses using stepwise variable selection with the Akaike information criterion. A random participant-specific effect will be included to account for correlation between visits in the same participant. The model-based average within-group change in cognition for each group will be computed for each cognitive outcome at both 3 and 6 months. This will allow us to rank the groups in terms of average within-person gains in executive function and episodic memory. We will conduct a composite hypothesis test to assess if the changes at 3 and 6 months differ by groups (similar to a one-factor ANOVA but accounting for repeated measures). In addition, the effect sizes of ACT, SOP training only, and cycling only for cognitive outcomes will also be calculated using standardized mean difference with 95% confidence interval (CI) [(*M*_int_ – *M*_control_ at later time) – (*M*_int_ – *M*_control_ at baseline)]/intra-participant standard deviation; “int” refers to ACT, SOP training only, or cycling only; “control” here refers to attention control. The effect size of ACT will be compared with the sum of the effect sizes of cycling only and SOP training only to determine the additive/synergistic effect.

#### Aim II. Examine the underlying mechanisms of ACT over 6 months

Aim II involves the following hypotheses 2a–2c:

*Hypothesis 2a*. Linear mixed-effects models similar to the ones developed in H1 will be fit for AD signature cortical thickness, functional connectivity in the DMN, and aerobic fitness. We will obtain the model-based estimates of the change in these mechanistic measures by randomized groups to determine which group had the largest change, and we will conduct a composite hypothesis test to assess if the changes in aerobic fitness at 3 and 6 months and in AD signature cortical thickness and DMN at 6 months differ significantly by group.

*Hypothesis 2b*. We will consider the same linear mixed-effects models as in H1 but will include changes in AD signature cortical thickness, functional connectivity in the DMN, and aerobic fitness at the corresponding time point in the models. Because of likely collinearity in these measures, separate models will be fit for each measure.

*Hypothesis 2c*. We will fit a longitudinal mixed-effects model (similar to H1) for the change in cognition, which will include both the change in aerobic fitness and the mediator’s changes in AD signature cortical thickness and functional connectivity, and longitudinal mixed-effects models for the changes in AD signature cortical thickness and functional connectivity that include aerobic fitness as a predictor. The estimated controlled direct effect and natural indirect effect can then be easily derived from the estimated coefficients of these models.

#### Aim III. Calculate the long-term effect sizes of ACT on cognition and clinical AD conversion to inform future phase III RCTs

Effect sizes for cognition and mechanisms will be calculated using effect-size equations similar to those proposed in Aim I. Time to AD conversion and reversion to normal from randomization will be compared among groups using log-rank tests. In addition, using the model developed in Aim I, we will compute the within-group model-based average changes for each cognitive outcome at 12 and 18 months for each group and conduct a composite hypothesis test to assess group differences in cognitive changes at 12 and 18 months.

### Ethics

This study was approved by the IRBs at both UMN and UR as well as by the DSMB. Any subsequent changes or amendments to the protocol will be submitted to the IRBs and DSMB for approval. Initial consent takes place during step 2, the in-person interview. The staff will assess the participant’s capacity to consent using the UCSD Brief Assessment of Capacity to Consent (UBACC) form modified for the ACT trial. A participant must score a 2 on items 1, 2, 4, 6, 7, and 9 on the assessment for inclusion in the study. If a participant scores less than 2 on any of these items, the staff will re-explain the study and then ask the participant to return on another day to re-take the UBACC. If the participant scores less than 2 on any of these items again, then the subject is not eligible to participate in the study.

Consenting will be an ongoing process during the study. Verbal consent will be obtained at the beginning of each activity session and during the monthly assessment of activity participation during the 1-year follow-up period. Signed paper consent will be obtained prior to subsequent data collections. The participants’ capacity to consent will be re-assessed at 12 and 18 months, prior to data collection, as in the in-person screening. Consent at data collection visits (12 and 18 months) will be conducted similarly to initial consent. Surrogate consent will be obtained for participants who lose the capacity to consent from their representative (i.e., caregiver), and assent from the participant will be noted on the form. Representatives will be identified in order of priority (e.g., durable power of attorney for health care, court-appointed guardian for health care decisions, spouse, and adult child). We will only follow up with the participants who provide consent/assent.

In the event that subjects suffered research-related injury, treatment will be available, including first aid, emergency treatment, and follow-up care as needed. However, care for such injuries during and after the study period will be billed in the ordinary manner to the subjects and their insurance companies.

### Dissemination

Findings from the study will be disseminated through publications and presentations. Participants and their family members will receive a copy of the published main findings. Authorship will follow the established publication guidelines such as those of the International Committee of Medical Journal Editors. We do not intend to use professional writers. Access to the final trial data set will follow the guidelines of the NIA.

## Discussion

The RCT is the gold standard design for determining the causal effect of an intervention on an outcome. Alternative designs include cross-over and usual care or waitlist as controls. Cross-over designs are ideal for interventions without carry-over effects, which is not the case for ACT. Usual-care or waitlist controls preclude assessing the placebo or Hawthorne effect of ACT.

A potential problem is unblinding. We have built in four strategies to ensure blinding: randomization using permuted blocks; all investigators blinded to group assignments; all participants blinded to the study aims and reminded as needed not to discuss their experiences with the outcome assessor; and outcome assessors blinded to the study aims, group assignments, and previous test results, and interacting with participants only for data collection [52]. Assessors will not interact with other staff who are not blinded (e.g., exercise interventionists) and will participate in separate meetings. Blinding success will be assessed after each data collection: Was group assignment unveiled? Why? For participants whose group assignments are revealed, a different blinded assessor will collect subsequent data. Other strategies include the use of standardized protocol, operations manual, forms, and reliable and valid variable measures. The analyses will incorporate all design features (e.g., site, repeated measures within persons, missing data).

While our aims are not focused on age and sex, we will take two measures to ensure adequate attention to them. First, we will stratify enrollment by age and target an enrollment of 60% women, because age and gender are established risk factors for AD [66]. Second, we will conduct an exploratory analysis examining whether age and sex moderate intervention effects on outcomes. We do not intend a fully powered analysis of this question for this RCT. However, large age/sex differences may be discernible and generate hypotheses for subsequent studies.

We do not plan to emphasize single-domain vs. multiple-domain MCI phenotype when identifying our sample given the change of emphasis in the updated NIA-Alzheimer’s Association (AA) criteria [3, 67]. However, we will secondarily examine the effect of the intervention on these phenotypes to specify a subsample who may benefit, particularly if the proposed intervention is effective for improving primary cognitive outcomes.

The multi-site design is advantageous compared to a single-site design to ensure timely enrollment, reduce regional influences on study implementation and results, increase sample representativeness, and enhance the generalizability of the study findings. A potential problem is differential implementation across our two sites. We have co-created the study protocol, materials, and operations manual and cross-trained investigators and staff via conference calls, video streaming, and site visits. We will implement additional strategies to ensure implementation consistency, including quarterly consistency checks, regular cross-site meetings, and treatment fidelity checks.

In summary, the ACT trial will be the first RCT to examine the additive/synergistic effects of a novel ACT intervention on cognition and relevant mechanisms in aMCI and to advance AD prevention research by providing precise immediate and long-term effect-size estimates of ACT to inform future fully powered, large-scale phase III RCTs.

## Trial status

The status of the trial at the time of manuscript submission is open for enrollment, and we expect enrollment accrual to complete in 2021.

## Abbreviations

ACT: Combined Aerobic exercise and Cognitive Training
AD: Alzheimer’s disease
ADAS-Cog: Alzheimer’s Disease Assessment Scale-Cognition
ADL-PI: Activities of Daily Living-Prevention Instrument
ADNI: Alzheimer’s Disease Neuroimaging Initiative
ANCOVA: Analysis of covariance
BOLD: Blood oxygen level-dependent (signal)
BVMT: Brief Visuospatial Memory Test
DSMB: Data Safety and Monitoring Board
FLAIR: Fluid-attenuated inversion recovery
HR: Heart rate
IADL: Instrumental activities of daily living
IRB: Institutional Review Board
ISWT: Incremental Shuttle Walk Test
MCI: Mild cognitive impairment
MLA: Mental leisure activity
MoCA: Montreal Cognitive Assessment
MPRAGE: Magnetization-prepared rapid gradient echo imaging
MRI: Magnetic resonance imaging
NIA: National Institute on Aging
NIH: National Institutes of Health
PASE: Physical Activity Scale for the Elderly
PSMS: Physical Self-Maintenance Scale
RA: Research assistant
RAVLT: Rey Auditory Verbal Learning Test
RCT: Randomized controlled trial
RPE: Rating of Perceived Exertion
SD: Standard deviation
SOP: Speed of processing
UBACC: UCSD Brief Assessment of Capacity to Consent
UMN: University of Minnesota
UR: University of Rochester
WHODAS: WHO Disability Assessment Schedule
WTAR: Wechsler Test of Adult Reading

## References

[1] Alzheimer's Association. 2018 Alzheimer's disease facts and figures. https://www.alz.org/alzheimers-dementia/facts-figures. Accessed 14 Mar 2018.

[2] Hebert LE, Weuve J, Scherr PA, Evans DA (2013). Alzheimer disease in the United States (2010–2050) estimated using the 2010 census. Neurology.

[3] Albert MS, DeKosky ST, Dickson D (2011). The diagnosis of mild cognitive impairment due to Alzheimer’s disease: recommendations from the National Institute on Aging-Alzheimer’s Association workgroups on diagnostic guidelines for Alzheimer’s disease. Alzheimers Dement.

[4] Mitchell AJ, Shiri-Feshki M (2009). Rate of progression of mild cognitive impairment to dementia—-meta-analysis of 41 robust inception cohort studies. Acta Psychiatr Scand.

[5] Lin F, Heidrich SM (2012). Role of older adult’s illness schemata in coping with Mild Cognitive Impairment. J Psychosom Res.

[6] Daviglus ML, Bell CC, Berrettini W (2010). National Institutes of Health state-of-the-science conference statement: preventing Alzheimer’s disease and cognitive decline. Ann Intern Med.

[7] American College of Sports Medicine (2013). ACSM’s guidelines for exercise testing and prescription.

[8] Andel R, Crowe M, Pedersen NL, Fratiglioni L, Johansson B, Gatz M (2008). Physical exercise at midlife and risk of dementia three decades later: a population-based study of Swedish twins. J Gerontol A Biol Sci Med Sci.

[9] Fratiglioni L, Paillard-Borg S, Winblad B (2004). An active and socially integrated lifestyle in late life might protect against dementia. Lancet Neurol.

[10] Larson EB, Wang L, Bowen JD (2006). Exercise is associated with reduced risk for incident dementia among persons 65 years of age and older. Ann Intern Med.

[11] Laurin D (2001). Physical activity and risk of cognitive impairment and dementia in elderly persons. Arch Neurol.

[12] Rovio S, Kareholt I, Helkala EL (2005). Leisure-time physical activity at midlife and the risk of dementia and Alzheimer’s disease. Lancet Neurol.

[13] Hassmen P, Ceci R, Backman L (1992). Exercise for older women: a training method and its influences on physical and cognitive performance. Eur J Appl Physiol Occup Physiol.

[14] Hawkins H, Kramer A, Capaldi D (1992). Aging, exercise, and attention. Psychol Aging.

[15] Rikli RE, Edwards DJ (1991). Effects of a three-year exercise program on motor function and cognitive processing speed in older women. Res Q Exerc Sport.

[16] Williams P, Lord SR (1997). Effects of group exercise on cognitive functioning and mood in older women. Aust N Z J Public Health.

[17] van Uffelen JGZ, Chinapaw MJM, van Mechelen W, Hopman-Rock M (2008). Walking or vitamin B for cognition in older adults with mild cognitive impairment? A randomised controlled trial. Br J Sports Med.

[18] Zheng G, Xia R, Zhou W, Tao J, Chen L (2016). Aerobic exercise ameliorates cognitive function in older adults with mild cognitive impairment: a systematic review and meta-analysis of randomised controlled trials. Br J Sports Med.

[19] Lautenschlager NT, Cox KL, Flicker L (2008). Effect of physical activity on cognitive function in older adults at risk for Alzheimer disease: a randomized trial. J Am Med Assoc.

[20] Burns JM, Cronk BB, Anderson HS (2008). Cardiorespiratory fitness and brain atrophy in early Alzheimer disease. Neurology.

[21] Burns JM, Mayo MS, Anderson HS, Smith HJ, Donnelly JE (2008). Cardiorespiratory fitness in early-stage Alzheimer disease. Alzheimer Dis Assoc Disord.

[22] Bugg JM, Head D (2011). Exercise moderates age-related atrophy of the medial temporal lobe. Neurobiol Aging.

[23] Erickson KI, Prakash RS, Voss MW (2009). Aerobic fitness is associated with hippocampal volume in elderly humans. Hippocampus.

[24] Spartano NL, Himali JJ, Beiser AS (2016). Midlife exercise blood pressure, heart rate, and fitness relate to brain volume 2 decades later. Neurology.

[25] Burdette JH, Laurienti PJ, Espeland MA (2010). Using network science to evaluate exercise-associated brain changes in older adults. Front Aging Neurosci.

[26] Rosano C, Venkatraman VK, Guralnik J (2010). Psychomotor speed and functional brain MRI 2 years after completing a physical activity treatment. J Gerontol A Biol Sci Med Sci.

[27] Voss MW, Prakash RS, Erickson KI (2010). Plasticity of brain networks in a randomized intervention trial of exercise training in older adults. Front Aging Neurosci.

[28] Voss MW, Erickson KI, Prakash RS (2010). Functional connectivity: a source of variance in the association between cardiorespiratory fitness and cognition?. Neuropsychologia.

[29] Martin M, Clare L, Altgassen AM, Cameron MH, Zehnder F (2011). Cognition-based interventions for healthy older people and people with mild cognitive impairment. Cochrane Database Syst Rev.

[30] Kueider AM, Parisi JM, Gross AL, Rebok GW (2012). Computerized cognitive training with older adults: a systematic review. PLoS One.

[31] Lovden M, Backman L, Lindenberger U, Schaefer S, Schmiedek F (2011). A theoretical framework for the study of adult cognitive plasticity. Psychol Bull.

[32] Valdes EG, O’Connor ML, Edwards JD (2012). The effects of cognitive speed of processing training among older adults with psychometrically- defined mild cognitive impairment. Curr Alzheimer Res.

[33] Mozolic JL, Hayasaka S, Laurienti PJ (2012). A cognitive training intervention increases resting cerebral blood flow in healthy older adults. Front Hum Neurosci.

[34] Takeuchi H, Taki Y, Hashizume H, et al. Effects of training of processing speed on neural systems. J Neurosci. 2011;`31(34):12139–48. 10.1523/JNEUROSCI.2948-11.2011 PMC6623215 21865456

[35] Lin F, Heffner KL, Ren P (2016). Cognitive and neural effects of vision-based speed-of-processing training in older adults with amnestic mild cognitive impairment: a pilot study. J Am Geriatr Soc.

[36] Curlik DM, Shors TJ (2013). Training your brain: do mental and physical (MAP) training enhance cognition through the process of neurogenesis in the hippocampus?. Neuropharmacology.

[37] Fabel K, Wolf SA, Ehninger D, Babu H, Leal-Galicia P, Kempermann G (2009). Additive effects of physical exercise and environmental enrichment on adult hippocampal neurogenesis in mice. Front Neurosci.

[38] Kempermann G (2002). Neuroplasticity in old age: sustained fivefold induction of hippocampal neurogenesis by long-term environmental enrichment. Ann Neurol.

[39] Galvan V, Bredesen DE (2007). Neurogenesis in the adult brain: implications for Alzheimer’s disease. CNS Neurol Disord Drug Targets.

[40] Hotting K, Roder B (2013). Beneficial effects of physical exercise on neuroplasticity and cognition. Neurosci Biobehav Rev.

[41] Shors TJ, Anderson ML, Curlik DM, Nokia MS (2012). Use it or lose it: how neurogenesis keeps the brain fit for learning. Behav Brain Res.

[42] Barnes DE, Santos-Modesitt W, Poelke G (2013). The Mental Activity and eXercise (MAX) trial: a randomized controlled trial to enhance cognitive function in older adults. JAMA Intern Med.

[43] McDaniel MA, Binder EF, Bugg JM (2014). Effects of cognitive training with and without aerobic exercise on cognitively demanding everyday activities. Psychol Aging.

[44] Fabre C, Chamari K, Mucci P, Masse-Biron J, Prefaut C (2002). Improvement of cognitive function by mental and/or individualized aerobic training in healthy elderly subjects. Int J Sports Med.

[45] Legault C, Jennings JM, Katula JA (2011). Designing clinical trials for assessing the effects of cognitive training and physical activity interventions on cognitive outcomes: the Seniors Health and Activity Research Program Pilot (SHARP-P) study, a randomized controlled trial. BMC Geriatr.

[46] Theill N, Schumacher V, Adelsberger R, Martin M, Jancke L (2013). Effects of simultaneously performed cognitive and physical training in older adults. BMC Neurosci.

[47] Train the Brain Consortium (2017). Randomized trial on the effects of a combined physical/cognitive training in aged MCI subjects: the Train the Brain study. Sci Rep.

[48] Onken LS, Carroll KM, Shoham V, Cuthbert BN, Riddle M (2014). Reenvisioning clinical science: unifying the discipline to improve the public health. Clin Psychol Sci.

[49] Jack CR, Wiste HJ, Weigand SD (2015). Different definitions of neurodegeneration produce similar amyloid/neurodegeneration biomarker group findings. Brain.

[50] Sorg C, Riedl V, Muhlau M (2007). Selective changes of resting-state networks in individuals at risk for Alzheimer’s disease. Proc Natl Acad Sci U S A.

[51] Schulz KF, Altman DG, Moher D (2010). CONSORT 2010 statement: updated guidelines for reporting parallel group randomised trials. Ann Intern Med.

[52] Chan AW, Tetzlaff JM, Altman DG (2013). SPIRIT 2013 statement: defining standard protocol items for clinical trials. Ann Intern Med.

[53] Yu F, Nelson NW, Savik K, Wyman JF, Dyskin M, Bronas UG (2013). Affecting cognition and quality of life via aerobic exercise in Alzheimer’s disease. West J Nurs Res.

[54] Lin F, Heffner K, Ren P, Tadin D (2017). A role of the parasympathetic nervous system in cognitive training. Curr Alzheimer Res.

[55] Salisbury D, Yu F (2018). Aerobic fitness and cognitive changes after exercise training in Alzheimer’s disease. J Exerc Clin Psychol.

[56] Kramer JH, Mungas D, Possin KL (2014). NIH EXAMINER: conceptualization and development of an executive function battery. J Int Neuropsychol Soc.

[57] Possin KL, Feigenbaum D, Rankin KP (2013). Dissociable executive functions in behavioral variant frontotemporal and Alzheimer dementias. Neurology.

[58] Estevez-Gonzalez A, Kulisevsky J, Boltes A, Otermin P, Garcia-Sanchez C (2003). Rey verbal learning test is a useful tool for differential diagnosis in the preclinical phase of Alzheimer’s disease: comparison with mild cognitive impairment and normal aging. Int J Geriatr Psychiatry.

[59] Gale SD, Baxter L, Thompson J (2016). Greater memory impairment in dementing females than males relative to sex-matched healthy controls. J Clin Exp Neuropsychol.

[60] Singh SJ, Morgan MD, Scott S, Walters D, Hardman AE (1992). Development of a shuttle walking test of disability in patients with chronic airways obstruction. Thorax.

[61] McKhann GM, Knopman DS, Chertkow H (2011). The diagnosis of dementia due to Alzheimer’s disease: recommendations from the National Institute on Aging and the Alzheimer’s Association workgroup. Alzheimers Dement.

[62] Jack CR, Barnes J, Bernstein MA (2015). Magnetic resonance imaging in Alzheimer’s Disease Neuroimaging Initiative 2. Alzheimers Dement.

[63] Yu F, Bronas UG, Konety S (2014). Effects of aerobic exercise on cognition and hippocampal volume in Alzheimer’s disease: study protocol of a randomized controlled trial (The FIT-AD trial). Trials.

[64] Bellg AJ, Borrelli B, Resnick B, et al. Ehancing treatment fidelity in health behavior change studies: Best practices and recommendations from the NIH behavior change consortium. Health Psychol. 2004;23(5):443–51. 10.1037/0278-6133.23.5.443 15367063

[65] Yu F (2013). Improving recruitment, retention, and adherence to 6-month cycling in Alzheimer’s disease. Geriatr Nurs.

[66] Lindsay J, Laurin D, Verreault R (2002). Risk factors for Alzheimer’s disease: a prospective analysis from the Canadian Study of Health and Aging. Am J Epidemiol.

[67] Jack CR, Bennett DA, Blennow K (2018). NIA-AA Research Framework: toward a biological definition of Alzheimer’s disease. Alzheimers Dement.

